# A Novel Red-Emitting Na_2_NbOF_5_:Mn^4+^ Phosphor with Ultrahigh Color Purity for Warm White Lighting and Wide-Gamut Backlight Displays

**DOI:** 10.3390/ma14185317

**Published:** 2021-09-15

**Authors:** Jingshan Hou, Wenxiang Yin, Langping Dong, Yang Li, Yufeng Liu, Zhifu Liu, Guoying Zhao, Ganghua Zhang, Yongzheng Fang

**Affiliations:** 1 Shanghai Institute of Technology, School of Materials Science and Engineering, Shanghai 201418, China; yinwenxiang1996@163.com (W.Y.); liyang123@sit.edu.cn (Y.L.); yfliu@mail.sitp.ac.cn (Y.L.); liuzf@sit.edu.cn (Z.L.); zhaogy135@126.com (G.Z.); zhangjiesss923@163.com (G.Z.); 2 Key Laboratory of Infrared Imaging Materials and Detectors, Shanghai Institute of Technical Physics, Chinese Academy of Sciences, 500 Yu Tian Road, Shanghai 200083, China; 3 Shanghai Key Laboratory of Engineering Materials Application and Evaluation, Shanghai Research Institute of Materials, Shanghai 200437, China

**Keywords:** Mn^4+^-doped red phosphors, oxyfluoride, white LEDs, high color purity, wide-gamut backlight displays

## Abstract

In this work, a novel red-emitting oxyfluoride phosphor Na_2_NbOF_5_:Mn^4+^ with an ultra-intense zero-phonon line (ZPL) was successfully synthesized by hydrothermal method. The phase composition and luminescent properties of Na_2_NbOF_5_:Mn^4+^ were studied in detail. The photoluminescence excitation spectrum contains two intense excitation bands centered at 369 and 470 nm, which match well with commercial UV and blue light-emitting diode (LED) chips. When excited by 470 nm blue light, Na_2_NbOF_5_:Mn^4+^ exhibits red light emission dominated by ZPL. Notably, the color purity of the Na_2_NbOF_5_:Mn^4+^ red phosphor can reach 99.9%. Meanwhile, the Na_2_NbOF_5_:Mn^4+^ phosphor has a shorter fluorescence decay time than commercial K_2_SiF_6_:Mn^4+^, which is conducive to fast switching of images in display applications. Profiting from the intense ZPL, white light-emitting diode (WLED) with high color rendering index of Ra = 86.2 and low correlated color temperature of T_c_ = 3133 K is realized using yellow YAG:Ce^3+^ and red Na_2_NbOF_5_:Mn^4+^ phosphor. The WLED fabricated using CsPbBr_3_ quantum dots (QDs) and red Na_2_NbOF_5_:Mn^4+^ phosphor shows a wide color gamut of 127.56% NTSC (National Television Standard Committee). The results show that red-emitting Na_2_NbOF_5_:Mn^4+^ phosphor has potential application prospects in WLED lighting and display backlight.

## 1. Introduction

High luminescence efficiency, environmentally friendly features and long operating lifetimes are all advantageous performance aspects of white light-emitting diodes (WLEDs), which have obtained widespread attention. WLEDs have been widely used in solid-state illumination and liquid crystal display backlight [1,2,3,4,5,6]. At present, the wide color gamut WLED backlight is mainly composed of blue InGaN chip, K_2_SiF_6_:Mn^4+^ red phosphor and green *β*-SiALON:Eu^2+^ phosphor [7,8,9]. However, the long decay time (~8 ms) of K_2_SiF_6_:Mn^4+^ (KSF:Mn^4+^) red phosphor easily affects the image-retention performance of fast-response backlight displays [10,11,12]. Apparently, phosphor for LED backlight should not only possess a broad excitation band appropriate to LED chip emission and narrow band emission with high color purity, but also have appropriate decay time [13]. Therefore, the exploration of novel red-emitting phosphors with high color purity and short fluorescence lifetime for backlight displays is necessary.

For solid-state lighting, the commercial WLED is phosphor-converted light-emitting diode (LED) fabricated by a combination of InGaN chip and YAG:Ce^3+^ phosphor. However, due to the lack of red component, this type of WLED exhibits a cold white light emission with low color-rendering index (CRI, Ra < 80) and high correlated color temperature (CCT, T_c_ > 4500 K). It is thus clear that red phosphor is an important part of assembling high-CRI light sources. Presently, non-rare-earth Mn^4+^ red phosphors have been used in the packaging of WLEDs due to their high luminous efficacy and low cost [14,15,16]. Under UV or blue light excitation, Mn^4+^-doped oxide phosphors can emit a moderate-intensity deep red light in the range of 650–720 nm [17,18,19,20,21,22]. Fluoride phosphors (e.g., A_2_BF_6_:Mn^4+^; A = Na, K, Rb, Cs; B = Si, Ti, Ge) show strong red emission with high color purity around 630 nm [23,24,25,26]. The oxyfluoride compounds are regarded as succedaneous hosts for Mn^4+^ substitution because Mn^4+^ in some oxyfluoride hosts also presents parallel photoluminescent (PL) properties with Mn^4+^-activated fluoride phosphors. More interestingly, the oxyfluoride compounds may induce Mn^4+^ to exhibit excellent luminescence properties owing to distorted octahedral sites and F^-^ and O^2-^ mixed ligands [27]. Hence, the exploration for new Mn^4+^-doped red-emitting phosphors based on oxyfluorides is of great significance.

Recently, Mn^4+^-doped oxyfluoride red phosphors have been reported successively, such as ANaWO_2_F_4_:Mn^4+^ (A = Li, Na, K) [28], Na_2_WO_2_F_4_:Mn^4+^ [29], Cs_2_NbOF_5_:Mn^4+^ and Rb_2_NbOF_5_:Mn^4+^ [30,31]. However, to the best of our knowledge, the study on the luminescence properties of Na_2_NbOF_5_:Mn^4+^ has not been reported. Herein, we synthesized a novel red-emitting oxyfluoride phosphor Na_2_NbOF_5_:Mn^4+^ for the first time and systematically investigated its crystal structure, composition and PL properties. Finally, white LED for indoor lighting and backlight displays was packaged by employing the as-prepared Na_2_NbOF_5_:Mn^4+^ phosphor as a red supplement.

## 2. Experimental Section

### 2.1. Sample Preparation

The starting materials Nb_2_O_5_ (99.99%), NaF (A.R.), HF solution (40 wt%, A.R.), ethanol (AR, 95%) and methyl alcohol (AR, 99.5%) were used without any purification. K_2_MnF_6_ was obtained through an optimized route reported by Verstraete [32].

**Figure 1:**
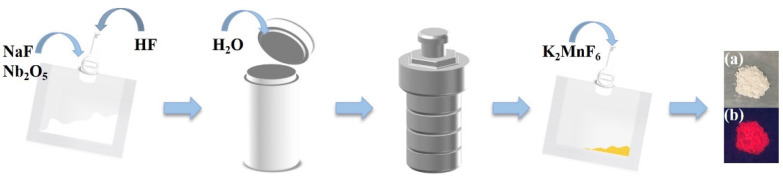
Schematic diagram of the experimental process for synthesizing NNOF:Mn^4+^ red-emitting phosphors, and digital photographs of the phosphor under (**a**) visible light, (**b**) 365 nm UV light.

The experimental process of synthesizing Na_2_NbOF_5_:*x*Mn^4+^ (abbreviated as NNOF:*x*Mn^4+^) red-emitting phosphors is shown in Figure 1. NaF (0.2688 g, 0.0064 mol), Nb_2_O_5_ (0.8058 g, 0.0032 mol) and 40% aqueous HF (2.88 mL) were added into a teflon pouch. Two pouches were placed in a 150 mL Teflon-lined stainless-steel autoclave filled with 50 mL deionized H_2_O as backfill and heated at 150 °C for 24 h, which were then slowly cooled to room temperature at 10 °C/h. Different amounts of K_2_MnF_6_ were dissolved in the solution with ultrasonic vibration until a light gold solution was formed in the pouch. To research the effect of the concentration of Mn^4+^ on the obtained phosphors, a series of NNOF:*x*Mn^4+^ samples with different concentrations of Mn^4+^ were prepared using the same method according to parameters listed in Table 1. After that, 5 mL of methanol was slowly injected into the pouch to obtain precipitation. The precipitate was further washed with ethanol, centrifuged three times to remove impurities and then dried in an oven at 60 °C for 3 h.

### 2.2. Characterization

The phase purity of the as-prepared samples was initially identified by taking X-ray diffraction (XRD) measurements from a X-ray powder diffractometer (Ultima IV-185, Tokyo, Japan) with Cu Kα radiation (*λ* = 1.5406 Å). The diffraction patterns were scanned at a scanning speed of 8°/min in the 2*θ* range from 10° to 80°. The infrared (IR) data was monitored by Fourier Transform Infrared Spectrometer (Bruker Tensor 27, Karlsruhe, Germany). The photoluminescence excitation (PLE) and emission (PL) spectra were obtained via a spectrophotometer (F-7000, HITACHI, Tokyo, Japan). Diffuse reflection spectrum was obtained using the spectrometer (Cary-5000, Varian, Palo Alto, CA, USA). The luminescence decay curve was recorded by a spectrometer (FS5, Edinburgh, UK). The morphology and elemental composition of the product were obtained by a scanning electron microscopy (SEM, JEOL JSM-6510, Tokyo, Japan) with an energy-dispersive spectrometer (EDS).

## 3. Results and Discussion

### 3.1. X-ray Diffraction and Structure Analysis

**Figure 2:**
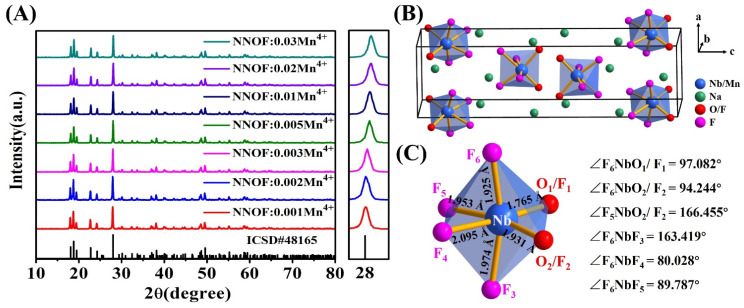
(**A**) XRD patterns of NNOF:*x*Mn^4+^ and enlarged XRD patterns in 2θ region of 27.5–28.5°; (**B**) Crystal structure scheme of NNOF:Mn^4+^; (**C**) Demonstration of the distorted [NbOF_5_]^2−^ octahedron.

Figure 2A shows the XRD patterns of Na_2_NbOF_5_:Mn^4+^ (NNOF:Mn^4+^) red phosphors doped with different doping amounts of Mn^4+^ and the enlarged XRD patterns in 2θ region of 27.5–28.5°. All the diffraction peaks of the samples matched with the Na_2_NbOF_5_ standard card (ICSD-48165, space group Pcnb (60), a = 5.089(1) Å, b = 5.512(1) Å, c = 18.207(4) Å, cell volume V = 510.72(18) Å^3^) and no impurity phase was found. The main diffraction peak moved to a higher angle with the increase in Mn content. According to Bragg’s diffraction law, the diffraction peak will move to a higher angle when small ions replace large ions into the lattice. The result indicated that the smaller Mn^4+^ (r = 0.53 Å, CN = 6) replaced the larger Nb^5+^ (r = 0.64 Å, CN = 6) into the lattice. However, when tetravalent Mn replaces pentavalent Nb into the lattice, charge mismatch occurs. A positive charge is required in the structure to maintain electrical neutrality. Positively charged oxygen vacancies are most likely to appear in the structure. This possible charge compensation can be represented by the following equation according to the Kröger–Vink notation [30]:

(1) $$K_{2}{\mathrm{MnF}}_{6}\;\overset{{\mathrm{Na}}_{2}{\mathrm{NbOF}}_{5}}{\to }\;2K_{\mathrm{Na}}^{\times }+{\mathrm{Mn}}_{\mathrm{Nb}}^{\prime }+V_{O}^{••}+5F_{F}^{\times }+{\;F}_{i}^{\prime }$$

where ${\mathrm{Mn}}_{\mathrm{Nb}}^{\prime }$ is the negative charge defect produced by the substitution of Nb^5+^ with Mn^4+^, $V_{O}^{••}$ is the oxygen vacancy and ${\;F}_{i}^{\prime }$ is the fluorine interstitial ion. The charge-balance is achieved by fluorine interstitial ion and oxygen vacancy.

Figure 2B depicts the simulated structure of the NNOF unit cell, where six twisted [NbOF_5_]^2−^ octahedra are regularly distributed in the cell. Figure 2C clearly depicts the coordination environment surrounding Nb. It is noticeable that Nb^5+^ coordinates six O^2−^/F^−^ to form a distorted [NbOF_5_]^2−^ octahedron, and the bond lengths of Nb-O_1_/F_1_, Nb-O_2_/F_2_, Nb-F_3_, Nb-F_4_, Nb-F_5_, Nb-F_6_ bonds are 1.765, 1.931, 1.974, 2.095, 1.953, 1.925 Å, respectively. At the same time, each bond angle of the [NbOF_5_]^2−^ octahedron is significantly different from the ideal bond angle (90°) of the regular octahedron.

**Figure 3:**
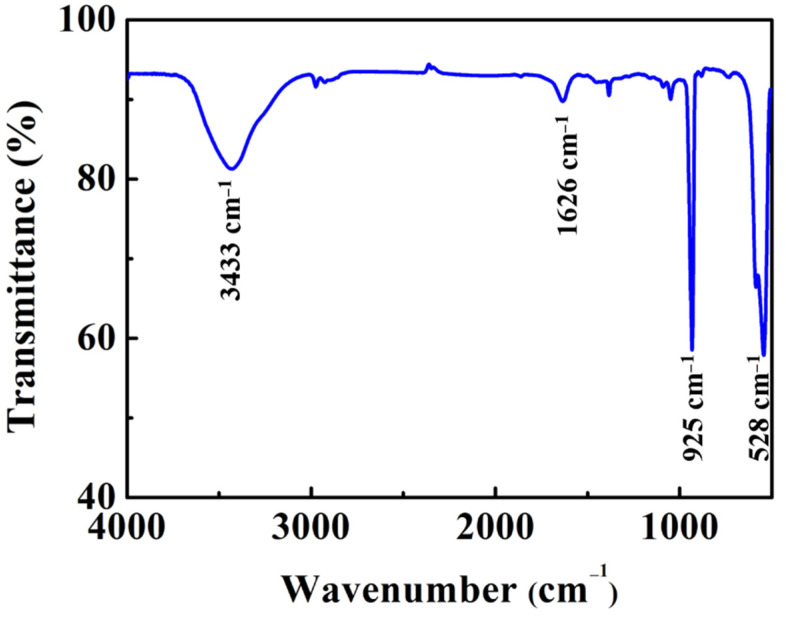
Infrared spectrum of NNOF:Mn^4+^.

Figure 3 shows the IR spectrum of NNOF:Mn^4+^ at room temperature. The wide band at 3433 cm^−1^ is due to the vibration of the O–H bonds, and the small peak at 1626 cm^−1^ is attributable to the bending vibration of the O–H bonds in the water adhering to the surface of the NNOF:Mn^4+^ minute particles. The IR spectrum shows two strong sharp peaks at 925 and 528 cm^−1^, which are consistent with the Nb–O and Nb–F bonds in the structure, respectively [33].

### 3.2. Morphology and Composition Identification

**Figure 4:**
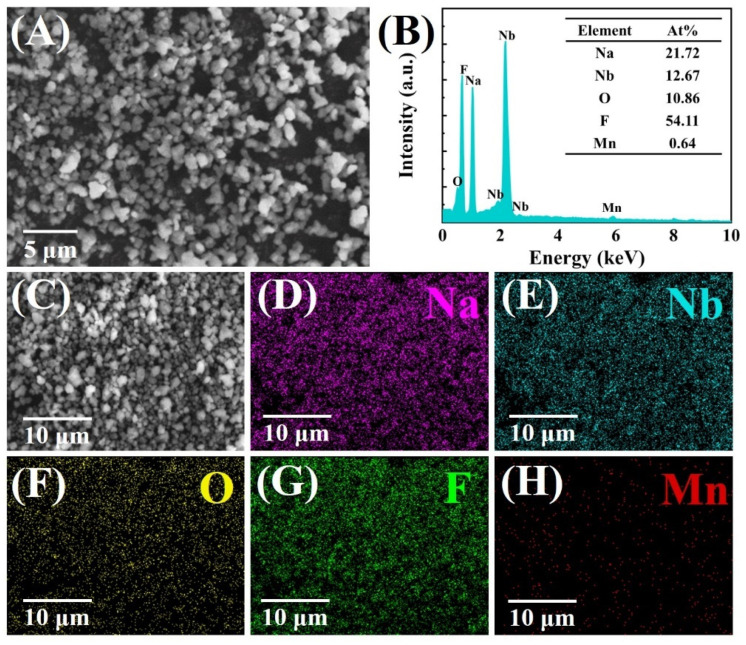
(**A**) SEM image; (**B**) EDS spectrum of NNOF:Mn^4+^ phosphor; (**C**–**H**) element mapping of Na, Nb, O, F and Mn in a selected area of NNOF:Mn^4+^ sample.

Figure 4A exhibits the SEM image of NNOF:Mn^4+^ phosphor. The obtained powder is composed of the irregular particle with clear edges and corners, indicating good crystallization of the sample. As shown in the EDS spectrum (Figure 4B), NNOF:Mn^4+^ red phosphor is composed of Na, Nb, O, F and Mn elements. The small amount of Mn in the test results indicates that Mn^4+^ has been successfully doped into the NNOF matrix. The atom percentages of Na, Nb, O and F are 21.72%, 12.67%, 10.86% and 54.11%, respectively, which are close to the stoichiometric ratio of 2:1:1:5 in the matrix. These data further confirmed the successful preparation of NNOF:Mn^4+^ phosphor. In Figure 4C–H, the EDS element mapping chart further proved the existence and uniform distribution of Na, Nb, O, F and Mn elements, and further confirmed the composition of NNOF:Mn^4+^.

### 3.3. Photoluminescence Properties

**Figure 5:**
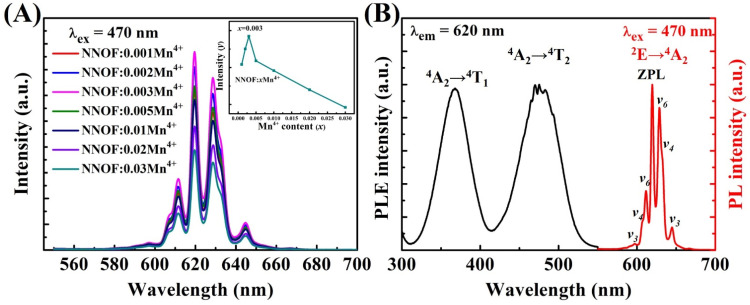
(**A**) PL spectra of NNOF:*x*Mn^4+^ (*x* = 0.001–0.03), the inset shows PL intensity of NNOF:*x*Mn^4+^ (*x* = 0.001–0.03) as a function of Mn^4+^ content; (**B**) PL and PLE spectra of NNOF:0.003Mn^4+^, ^4^T_1_, ^4^T_2_ and ^2^E are the three excited states and ^4^A_2_ is the ground state of Mn^4+^, *v_3_*–*v_6_* are the Stokes/anti-Stokes phonon sidebands and ZPL is the zero phonon line.

The emission spectra of NNOF:Mn^4+^ phosphors with different Mn^4+^ concentrations are shown in Figure 5A. When the concentration is 0.003, the luminous intensity is at the highest value. Due to the effect of concentration quenching, the luminous intensity of phosphor decreases with the increasing of Mn^4+^ concentration [34].

The red phosphor excited by blue chip used in the WLED requires a wide absorption in the blue region and an effective emission near the ideal red light within 650 nm. Figure 5B shows the PLE and PL spectra of NNOF:Mn^4+^ at room temperature. Obviously, two intense excitation bands centered at 369 (27,100 cm^−1^) and 470 nm (21,277 cm^−1^) can be observed in the excitation spectrum, which are caused by the spin allowed ^4^A_2_ → ^4^T_1_ and ^4^A_2_ → ^4^T_2_ transitions of the Mn^4+^ ions, respectively [35,36]. Under 470 nm excitation, NNOF:Mn^4+^ exhibits a narrow peak emission distributed between 575 and 675 nm. The results of excitation and emission spectra attested that the prepared NNOF:Mn^4+^ samples can be excited by blue light effectively and produce effective red emission. Meanwhile, the NNOF:0.003Mn^4+^ exhibits photoluminescence quantum yields (PLQYs) of 68.3% under 470 nm blue light excitation. The PLQY was obtained according to the method found in the reported work [37]. Notably, the intensity of zero-phonon line (ZPL) emission is higher than that of phonon sideband, which is different from most of previously reported emission spectra of Mn^4+^. The sharp ZPL emission peaking at 620 nm and Stokes/anti-Stokes phonon sidebands are derived from the coupling of antisymmetric *v_3_*, *v_4_*, and *v_6_* to the ZPL. The emission spectrum of Mn^4+^ doped phosphor is usually dominated by the anti-Stokes/Stokes phonon sideband, and the vibronic transition *v_6_* is always at the highest peak in the PL spectrum. Meanwhile, the ZPL of Mn^4+^:^2^E → ^4^A_2_ is generally very weak. Interestingly, the intensity of ZPL in the emission spectrum of NNOF:Mn^4+^ is higher than that of the *v_6_* sideband. Herein, the low symmetry of the Mn^4+^ center is considered to be the main reason for the intense ZPL in NNOF:Mn^4+^ [29,38,39]. The ultra-high ZPL emission is conducive to improving the color purity of red phosphors [40].

**Figure 6:**
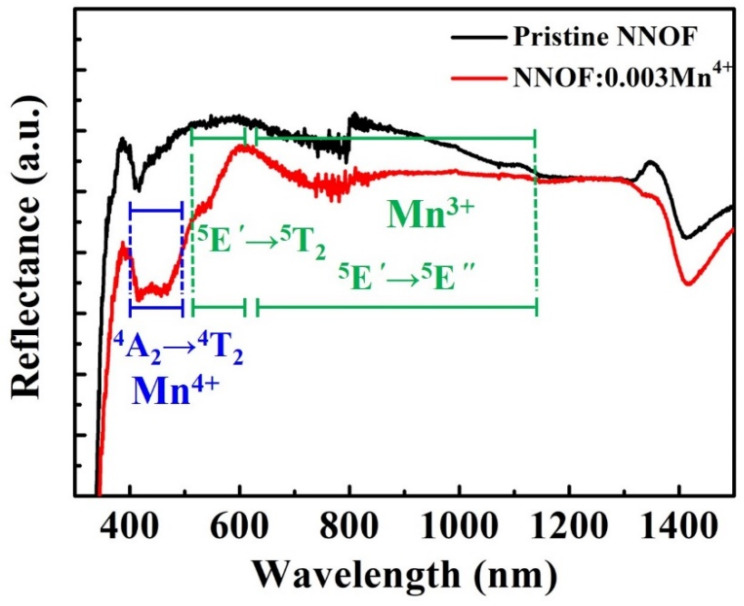
Diffuse reflection spectra of pristine NNOF and NNOF:0.003Mn^4+^, ^4^A_2_ and ^4^T_2_ are the ground state and excited state of Mn^4+^, respectively, ^5^E′ and ^5^E″ are two Jahn-Teller split ^5^E ground states and ^5^T_2_ is the excited state of Mn^3+^.

Figure 6 shows the diffuse reflectivity spectra of pristine NNOF and NNOF:0.003Mn^4+^ phosphor, from which it can be observed that the NNOF:0.003Mn^4+^ phosphor has an absorption band at 470 nm corresponding to the ^4^A_2_ → ^4^T_2_ electron transition of Mn^4+^. Due to the strong intrinsic absorption of pristine NNOF, the absorption band near 369 nm, which responds to the ^4^A_2_ → ^4^T_1_ electron transition of Mn^4+^, was completely covered. Compared with Mn^4+^ and Mn^2+^, Mn^3+^ is rarely encountered in the literature about luminescence. The Mn^3+^ ion has the corresponding characteristic absorption band due to the 3d^4^ configuration. In Figure 6, the ^5^E′ → ^5^T_2_ and ^5^E′ → ^5^E″ absorption bands of Mn^3+^ can be clearly observed in the 500–1100 nm range. The presence of Mn^3+^ impurity ions will lead to the reduction of quantum efficiency of phosphor [32].

### 3.4. Decay Curves, Chromaticity Coordinates (CIE) and Color Purity

**Figure 7:**
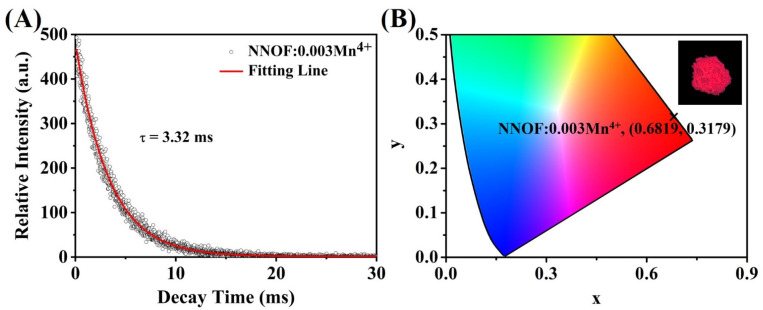
(**A**) Photoluminescence decay curve of NNOF:0.003Mn^4+^; (**B**) CIE coordinates of the NNOF:0.003Mn^4+^, inset: photo of phosphor illuminated by 365 UV lamp.

The decay time of phosphor is non-negligible in the application of display backlight. Long decay time phosphors may cause a certain degree of lag in image conversion. Figure 7A shows the photoluminescence decay curve of NNOF:0.003Mn^4+^ phosphor. The data of the luminescence decay curve conforms to the mono-exponential decay mode, as shown in the following formula:

(2) $$I(t)\;=\;I_{0}+A\;\exp(-\frac{t}{\tau })$$

where *I*_0_ and *I*(*t*) are the initial luminous intensity and the luminous intensity at time *t*, respectively, and *τ* represents the fluorescent lifetime. *τ* is then calculated to be 3.32 ms. The millisecond scale of the lifetime indicates that Mn^4+^ ions present forbid transitions in the intra-d-shell [8]. The NNOF:0.003Mn^4+^ phosphor with short fluorescence decay time (<5 ms) will be a hopeful red component for fast-response backlight displays. Herein, the decay time of NNOF:0.003Mn^4+^ is shorter than that of K_2_SiF_6_:Mn^4+^. To specify the reason for the shorter life of NNOF:0.003Mn^4+^, the distortion of the coordination polyhedron (DI) is calculated. The distortion degree of polyhedron is characterized by bond angle variance (*σ*^2^) and mean quadratic elongation (*λ*), which can be determined by following formula [29]:

(3) $$\sigma^{2}=\frac{1}{6}\sum_{i=1}^{6}{\left(l_{i}/l_{0}\right)}^{2}$$

(4) $$\lambda =\frac{1}{11}\sum_{i=1}^{12}{\left(\theta_{i}/\theta_{0}\right)}^{2}$$

where *l*_0_ is the distance from the center to the vertex of the regular octahedron with the same volume as the octahedron structure, *l*_i_ is the bond length of the studied octahedron, *θ*_0_ is the ideal bond angle (90°) of the regular octahedron and *θ*_i_ is the bond angle of the twisted octahedron. The corresponding values are shown in Table 2. The results indicate that the coordination environment of Mn^4+^ in NNOF:*x*Mn^4+^ is extremely distorted compared to K_2_SiF_6_:Mn^4+^. In fact, it is understandable that NNOF has a higher degree of distortion since the anion coordinated with the cation is mixed-anion with unequal radius. It has been reported that Mn^4+^ exhibits good luminescence properties in a highly symmetric structure [28]. Nevertheless, mixed anion coordination offers more possibilities for luminescent behavior. We believe that the rapid decay may be due to the low symmetry of Mn^4+^.

The CIE chromaticity coordinate of NNOF:0.003Mn^4+^ sample is shown in Figure 7B. It is observed that the prepared NNOF:0.003Mn^4+^ red phosphor emits a strong red light under the excitation of 365 nm ultraviolet lamp. The CIE chromaticity coordinates are (0.6819, 0.3179). To further understand the chromatic behaviors of the phosphor, its color purity was found by using the following formula [41]:

(5) $$\mathrm{Color}\;\mathrm{purity}=\frac{\sqrt{{(x\;-\;x_{i})}^{2}-\;(y\;-{\;y}_{i}{)}^{2}}}{\sqrt{{{(x}_{d}{\;-\;x}_{i})}^{2}{\;-\;(y}_{d}\;-\;y_{i}{)}^{2}}}\;\times \;100\%$$

Hereon, (*x*, *y*) represents the CIE coordinates of NNOF:0.003Mn^4+^ red phosphor, (*x*_i_, *y*_i_) represents the chromaticity coordinate of the equal-energy white light source with the value of (0.3333, 0.3333), and (*x*_d_, *y*_d_) stands for the CIE coordinates of the corresponding dominant wavelength of the illuminant. The calculated color purity of NNOF:0.003Mn^4+^ phosphor is about 99.9%, which is higher than the reported color purity of Mn^4+^-doped red phosphors, such as Cs_2_NbOF_5_:Mn^4+^ (99%) [30], K_2_LiAlF_6_:Mn^4+^ (89%) and K_2_NaAlF_6_:Mn^4+^ (97%) [42,43]. Obviously, the stronger ZPL emission can bring about a higher color purity. NNOF:Mn^4+^ red phosphor is very suitable for the application in LED backlight due to its ultra-high color purity.

### 3.5. Electroluminescence (EL) Performance of the Packaged WLEDs

**Figure 8:**
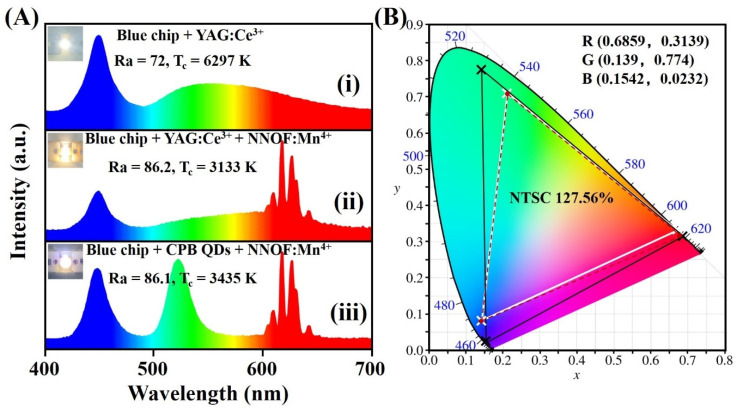
(**A**) Electroluminescence spectra of the WLED fabricated by (i) YAG:Ce^3+^, (ii) the mixture of YAG:Ce^3+^ with NNOF:Mn^4+^ and (iii) the mixture of CPB QDs with NNOF:Mn^4+^; insets exhibit the corresponding luminescent images of the packaged WLEDs; (**B**) Color gamut of the NTSC standard (white triangle), color gamut of the prepared NNOF:0.003Mn^4+^ phosphor matched with the standard blue and green coordinates defined by NTSC (red dotted triangle) and color gamut of the constructed WLED using CPB QDs and NNOF:Mn^4+^ (black triangle).

Figure 8A shows the EL spectra and photographs of the packaged WLEDs. Curve (i) is the spectrum of WLED produced by YAG:Ce^3+^ phosphor coupled with InGaN blue chip (3 V, 20 mA), and curve (ii) is the EL spectrum of WLED with the addition of NNOF:Mn^4+^ red phosphor as a contrast. Compared with the scheme of blue chip + YAG:Ce^3+^ (Ra = 72, CCT = 6297 K), the LED device with added NNOF:Mn^4+^ red component emits high-brightness warm white light (Ra = 86.2, CCT = 3133 K), indicating that the addition of NNOF:Mn^4+^ can improve the color rendering index (CRI) and correlated color temperature (CCT). Even more to the point, the luminous efficiency of WLED using NNOF:Mn^4+^ as red component can reach as high as 106.05 lm/W.

As shown in Figure 8B, the white triangle region is the color gamut composed of standard red (0.67, 0.33), blue (0.21, 0.71) and green (0.14, 0.08) coordinates, which is defined by the National Television Standards Committee (NTSC). When the chromaticity coordinates (0.6819, 0.3179) of the prepared NNOF:0.003Mn^4+^ phosphor are matched with the standard blue and green coordinates, we can obtain a larger gamut with a calculated value of 102.63% NTSC as depicted in the red dotted triangle in Figure 8B. To prove the application potential of the synthesized NNOF:Mn^4+^ red phosphor in the field of LED backlight displays, the EL spectrum of the WLED constructed with green-emitting CsPbBr_3_ quantum dots (abbreviated as CPB QDs) and NNOF:Mn^4+^ red phosphor is shown in curve (iii) in Figure 8A. The color gamut of the produced WLED device is shown in the black frame in Figure 8B, which is calculated as 127.56% of the NTSC color gamut and overlaps with NTSC by 99.46%. These results show the prospect of the as-prepared NNOF:Mn^4+^ red phosphor for its application in the field of backlight displays.

## 4. Conclusions

A novel Mn^4+^ doped oxyfluoride phosphor was successfully synthesized by hydrothermal method. The prepared NNOF:Mn^4+^ red phosphor can be matched well with commercial UV and InGaN blue chips because of its wide excitation band in the near ultraviolet and blue regions. When excited by blue light, the sample exhibited ultra-intense ZPL emission at 620 nm. Remarkably, the color purity of NNOF:Mn^4+^ can reach as high as 99.9%. Moreover, the WLED fabricated by using NNOF:Mn^4+^ red phosphor and commercial YAG:Ce^3+^ produced warm white light emission with low CCT value of 3133 K, high Ra value of 86.2 and luminous efficiency of 106.05 lm/W. Finally, a white LED with a wide color gamut of 127.56% NTSC was packaged on a InGaN blue chip using NNOF:Mn^4+^ red phosphor and green-emitting CPB QDs. These results show that NNOF:Mn^4+^ red phosphor has potential application prospects in lighting or display backlights.

## Figures and Tables

**Table 1:** Synthesis parameters of NNOF:*x*Mn^4+^ phosphors with different doping amounts of Mn^4+^.

Samples	The Molar Quantities of K_2_MnF_6_ (mol)	Actual Doping Amount of Mn^4+^ (*x* mol)
1	3.2 × 10^−6^	0.001
2	6.4 × 10^−6^	0.002
3	9.6 × 10^−6^	0.003
4	1.6 × 10^−5^	0.005
5	3.2 × 10^−5^	0.01
6	6.4 × 10^−5^	0.02
7	9.6 × 10^−5^	0.03

**Table 2:** Polyhedral distortion index and corresponding zero-phonon line (ZPL) intensities in Na_2_NbOF_5_ and K_2_SiF_6_ matrices.

Compounds	*σ* ^2^	*λ*	ZPL Intensity
Na_2_NbOF_5_	43.6362	1.0184	Very strong
K_2_SiF_6_	0.0000	1.0000	Very weak

## Data Availability

Data sharing is not applicable to this article.

## References

[1] Nair G.B., Swart H.C., Dhoble S.J. (2020). A review on the advancements in phosphor-converted light emitting diodes (pc-LEDs): Phosphor synthesis device fabrication and characterization. Prog. Mater. Sci..

[2] Cho J., Park J.H., Kim J.K., Schubert E.F. (2017). White light-emitting diodes: History, progress, and future. Laser Photonics Rev..

[3] Xia Z., Xu Z., Chen M., Liu Q. (2016). Recent developments in the new inorganic solid-state LED phosphors. Dalton Trans..

[4] Chen D., Zhou Y., Zhong J. (2016). A review on Mn^4+^ activators in solids for warm white light-emitting diodes. RSC Adv..

[5] Wang S., Xu Y., Chen T., Jiang W., Liu J., Zhang X., Jiang W., Wang L. (2021). A red phosphor LaSc_3_(BO_3_)_4_:Eu^3+^ with zero-thermal-quenching and high quantum efficiency for LEDs. Chem. Eng. J..

[6] Xia Z., Meijerink A. (2017). Ce^3+^-Doped garnet phosphors: Composition modification, luminescence properties and applications. Chem. Soc. Rev..

[7] Wang L., Xie R.J., Suehiro T., Takeda T., Hirosaki N. (2018). Down-conversion nitride materials for solid state lighting: Recent advances and perspectives. Chem. Rev..

[8] Lin H., Hu T., Huang Q., Cheng Y., Wang B., Xu J., Wang J., Wang Y. (2017). Non-rare-earth K_2_*X*F_7_:Mn^4+^ (*X* = Ta, Nb): A highly-efficient narrow-band red phosphor enabling the application in wide-color-gamut LCD. Laser Photonics Rev..

[9] Wang L., Wang X., Kohsei T., Yoshimura K., Izumi M., Hirosaki N., Xie R. (2015). Highly efficient narrow-band green and red phosphors enabling wider color-gamut LED backlight for more brilliant displays. Opt. Express.

[10] Murphy J.E., Garcia-Santamaria F., Setlur A.A., Sista S. (2015). 62.4: PFS, K_2_SiF_6_:Mn^4+^: The Red-line Emitting LED Phosphor behind GE’s TriGain Technology™ Platform. SID Symp. Dig. Tech. Pap..

[11] Yu X., Wang Y. (2010). Synthesis and photoluminescence improvement of monodispersed Zn_2_SiO_4_:Mn^2+^ nanophosphors. J. Alloys Compd..

[12] Yadav R.S., Pandey S.K., Pandey A.C. (2011). BaAl_12_O_19_:Mn^2+^ green emitting nanophosphor for PDP application synthesized by solution combustion method and its Vacuum Ultra-Violet Photoluminescence Characteristics. J. Lumin..

[13] Liang Z., Yang Z., Tang H., Guo J., Yang Z., Zhou Q., Tang S., Wang Z. (2019). Synthesis, luminescence properties of a novel oxyfluoride red phosphor BaTiOF_4_:Mn^4+^ for LED backlighting. Opt. Mater..

[14] Fang M.H., Wu W.L., Jin Y., Lesniewski T., Mahlik S., Grinberg M., Brik M.G., Srivastava A.M., Chiang C.Y., Zhou W. (2018). Control of luminescence by tuning of crystal symmetry and local structure in Mn^4+^-activated narrow band fluoride phosphors. Angew. Chem. Int. Ed..

[15] Song E., Zhou Y., Yang X.B., Liao Z., Zhao W., Deng T., Wang L., Ma Y., Ye S., Zhang Q. (2017). Highly efficient and stable narrow-band red phosphor Cs_2_SiF_6_:Mn^4+^ for high-power warm white LED applications. ACS Photonics.

[16] Lv L., Chen Z., Liu G., Huang S., Pan Y. (2015). Optimized photoluminescence of red phosphor K_2_TiF_6_:Mn^4+^ synthesized at room temperature and its formation mechanism. J. Mater. Chem. C.

[17] Adachi S. (2018). Photoluminescence properties of Mn^4+^-activated oxide phosphors for use in white-LED applications: A review. J. Lumin..

[18] Lü W., Lv W., Zhao Q., Jiao M., Shao B., You H. (2014). A novel efficient Mn^4+^ activated Ca_14_Al_10_Zn_6_O_35_ phosphor: Application in red-emitting and white LEDs. Inorg. Chem..

[19] Wang B., Lin H., Huang F., Xu J., Chen H., Lin Z., Wang Y. (2016). Non-Rare-Earth BaMgAl_10–2*x*_O_17_:*x*Mn^4+^, *x*Mg^2+^: A Narrow-Band Red Phosphor for Use as a High-Power Warm w-LED. Chem. Mater..

[20] Zhou Z., Zheng J., Shi R., Zhang N., Chen J., Zhang R., Suo H., Goldys E.M., Guo C. (2017). Ab initio site occupancy and far-red emission of Mn^4+^ in cubic-phase La(MgTi)_1/2_O_3_ for plant cultivation. ACS Appl. Mater. Int..

[21] Kong L., Liu Y., Dong L., Zhang L., Qiao L., Wang W., You H. (2020). Enhanced red luminescence in CaAl_12_O_19_:Mn^4+^ via doping Ga^3+^ for plant growth lighting. Dalton Trans..

[22] Cao R., Liu X., Bai K., Chen T., Guo S., Hu Z., Xiao F., Luo Z. (2018). Photoluminescence properties of red-emitting Li_2_ZnSn_2_O_6_:Mn^4+^ phosphor for solid-state lighting. J. Lumin..

[23] Nguyen H.D., Lin C.C., Fang M.H., Liu R.S. (2014). Synthesis of Na_2_SiF_6_:Mn^4+^ red phosphors for white LED applications by co-precipitation. J. Mater. Chem. C.

[24] Wang Z., Liu Y., Zhou Y., Zhou Q., Tan H., Zhang Q., Peng J. (2015). Red-emitting phosphors Na_2_*X*F_6_:Mn^4+^ (*X* = Si, Ge, Ti) with high colour-purity for warm white-light-emitting diodes. RSC Adv..

[25] Wei L.L., Lin C.C., Fang M.H., Brik M.G., Hu S.F., Jiao H., Liu R.S. (2015). A low-temperature co-precipitation approach to synthesize fluoride phosphors K_2_*M*F_6_:Mn^4+^ (*M* = Ge, Si) for white LED applications. J. Mater. Chem. C.

[26] Zhu H., Lin C.C., Luo W., Shu S., Liu Z., Liu Y., Kong J., Ma E., Cao Y., Liu R.S. (2014). Highly efficient non-rare-earth red emitting phosphor for warm white light-emitting diodes. Nat. Commun..

[27] Zhou Y., Zhang S., Wang X., Jiao H. (2019). Structure and luminescence properties of Mn^4+^-activated K_3_TaO_2_F_4_ red phosphor for white LEDs. Inorg. Chem..

[28] Hu M., Liu Z., Xia Y., Zhang G., Fang Y., Liu Y., Zhao G., Hou J. (2020). The photoluminescence adjustment of red phosphors *A*NaWO_2_F_4_:Mn^4+^ (*A* = Li, Na, K) by suitable tolerance factor designing. J. Mater. Sci. Mater. Electron..

[29] Hu T., Lin H., Cheng Y., Huang Q., Xu J., Gao Y., Wang J., Wang Y. (2017). A highly-distorted octahedron with a *C*_2v_ group symmetry inducing an ultra-intense zero phonon line in Mn^4+^-activated oxyfluoride Na_2_WO_2_F_4_. J. Mater. Chem. C.

[30] Ming H., Zhang J., Liu L., Peng J., Du F., Ye X., Yang Y., Nie H. (2018). A novel Cs_2_NbOF_5_:Mn^4+^ oxyfluoride red phosphor for light-emitting diode devices. Dalton. Trans..

[31] Wang Z., Yang Z., Yang Z., Wei Q., Zhou Q., Ma L., Wang X. (2018). Red phosphor Rb_2_NbOF_5_: Mn^4+^ for warm white light-emitting diodes with a high color-rendering index. Inorg. Chem..

[32] Verstraete R., Sijbom H.F., Joos J.J., Korthout K., Poelman D., Detavernier C., Smet P.F. (2018). Red Mn^4+^-doped fluoride phosphors: Why purity matters. ACS Appl. Mater. Int..

[33] Marvel M.R., Pinlac R.A.F., Lesage J., Stern C.L., Poeppelmeier K.R. (2009). Chemical hardness and the adaptive coordination behavior of the d^0^ transition metal oxide fluoride anions. Z. Anorg. Allg. Chem..

[34] Park K., Hakeem D.A. (2017). Improved photoluminescence properties of BaAl_2_Si_2_O_8_:Eu^3+^, Tb^3+^ phosphors by doping Tb^3+^. Ceram. Int..

[35] Jin Y., Fang M.H., Grinberg M., Mahlik S., Lesniewski T., Brik M.G., Luo G.Y., Lin J.G., Liu R.S. (2016). Narrow red emission band fluoride phosphor KNaSiF_6_:Mn^4+^ for warm white light-emitting diodes. ACS Appl. Mater. Int..

[36] Senden T., van Dijk-Moes R., Meijerink A. (2018). Quenching of the red Mn^4+^ luminescence in Mn^4+^-doped fluoride LED phosphors. Light Sci. Appl..

[37] Cao Y., Zhang G., Fang Y., Yin X., Lin Y., Zhao G., Liu Y., Sun H., Huang F., Hou J. (2021). Tuning Coordination Environments of Dopants through Topochemical Reaction Enables Substantial Enhancement of Luminescence in Mn^4+^-Doped Perovskite. J. Phys. Chem. C.

[38] Huang D., Zhu H., Deng Z., Zou Q., Lu H., Yi X., Guo W., Lu C., Chen X. (2019). Moisture-Resistant Mn^4+^-Doped Core–Shell-Structured Fluoride Red Phosphor Exhibiting High Luminous Efficacy for Warm White Light-Emitting Diodes. Angew. Chem. Int. Ed..

[39] Donegan J.F., Glynn T.J., Imbusch G.F., Remeika J.P. (1986). Luminescence and fluorescence line narrowing studies of Y_3_Al_5_O_12_:Mn^4+^. J. Lumin..

[40] Zhou Q., Liang Z., Shi D., Wang Z., Wang K., Tang H., Milićević B., Wu M. (2020). Double sites occupancy of Mn^4+^ in Cs_2_NaAlF_6_ with enhanced photoluminescence for white light-emitting diodes. J. Alloys Compd..

[41] Zhang X., Tsai Y.T., Wu S.M., Lin Y.C., Lee J.F., Sheu H.S., Cheng B.M., Liu R.S. (2016). Facile atmospheric pressure synthesis of high thermal stability and narrow-band red-emitting SrLiAl_3_N_4_:Eu^2+^ phosphor for high color rendering index white light-emitting diodes. ACS Appl. Mater. Int..

[42] Zhu Y., Cao L., Brik M.G., Zhang X., Huang L., Xuan T., Wang J. (2017). Facile synthesis, morphology and photoluminescence of a novel red fluoride nanophosphor K_2_NaAlF_6_:Mn^4+^. J. Mater. Chem. C.

[43] Zhu Y., Liu Y., Brik M.G., Huang L., Xuan T., Wang J. (2017). Controlled morphology and improved photoluminescence of red emitting K_2_LiAlF_6_:Mn^4+^ nano-phosphor by co-doping with alkali metal ions. Opt. Mater..

